# Opportunities and challenges in studying non-coding RNAs using neural organoid models

**DOI:** 10.3389/fnmol.2026.1801891

**Published:** 2026-04-29

**Authors:** Po-Sung Chiang, Meng-Han Tsai, Pei-Shan Hou

**Affiliations:** 1 Institute of Anatomy and Cell Biology, National Yang Ming Chiao Tung University, Taipei, Taiwan; 2 School of Medicine, College of Medicine, National Yang Ming Chiao Tung University, Taipei, Taiwan; 3 Department of Neurology, Kaohsiung Chang Gung Memorial Hospital, Kaohsiung, Taiwan; 4 Doctoral Program of Clinical and Experimental Medicine, National Sun Yat-sen University, Kaohsiung, Taiwan; 5 Chang Gung University School of Medicine, Taoyuan, Taiwan; 6 Brain Research Center, National Yang Ming Chiao Tung University, Taipei, Taiwan; 7 Institute of Brain Science, National Yang Ming Chiao Tung University, Taipei, Taiwan

**Keywords:** circular RNA (circRNA), long non-coding (lnc) RNA, microRNA, neural organoid, non-coding RNA (ncRNA)

## Abstract

Recent advancements in whole-genome sequencing have identified non-coding RNAs (ncRNAs) as pivotal regulators in biological systems. However, investigating neural-related ncRNAs *in vitro* remains challenging due to their complex spatial expression patterns and regulatory networks, which are inadequately captured in traditional two-dimensional cultures. The three-dimensional (3D) neural organoids have recently evolved from stochastic assemblies to engineered, region-specific, and integrated systems, providing a high-fidelity platform to overcome these limitations. This review summarizes the current landscape of ncRNA research within neural organoid systems, including miRNAs, lncRNAs, circRNAs, and snRNAs. By highlighting key findings in neurodevelopmental modeling and disease simulation, we discuss the implications of using these 3D models to decode the non-coding genome and their potential for preclinical applications. These advancements collectively solidify neural organoids as an essential platform, linking the high complexity of the non-coding genome to the functional reality of human brain development and pathology.

## The transition from stochastic growth to guided regionalization in neural organoids

**FIGURE 1:**
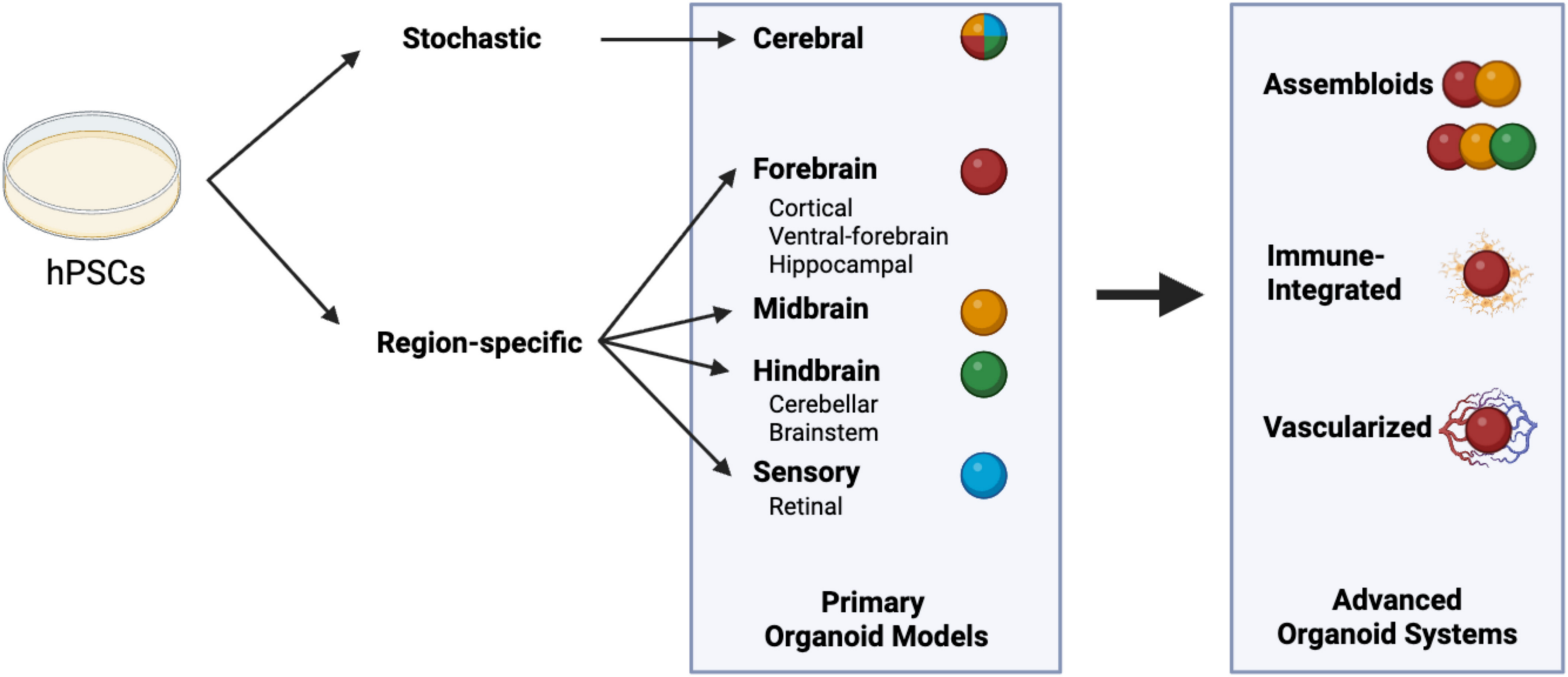
The classification and progression of neural organoid models. Neural organoids can be generated by two approaches: stochastic and region-specific differentiation protocols. In region-specific protocols, specific signaling molecules, such as morphogens, are utilized to pattern organoids into distinct regional identities, including the forebrain, midbrain, hindbrain, and sensory systems. These organoids can be further assembled into other organoids, integrated with immune cells, and cultured with endothelial cells to form an advanced system for studying the physiological complexity of the human brain. Created in BioRender. Chiang, P. (2026) https://BioRender.com/rhale74.

In recent decades, the emerging *in vitro* human neural organoid platform has undergone significant advancements, with increasing capability to simulate the complex structure and developmental timeline of the human brain. This high-fidelity organoid platform enables manipulation of precise spatiotemporal control. Two significant milestones were achieved, including a transition from stochastic, self-organizing cerebral assemblies to guided region-specific organoids and a critical change from solely neuronal constructs to integrated systems that incorporate essential non-neuronal lineages (Figure 1 and Table 1).

**TABLE 1:** Progression of neural organoids.

Organoid type	Major content	Year	References
Retinal organoids	Self-formation of optic cups producing stratified neural retina	2012	Eiraku et al., 2011; Nakano et al., 2012
Cerebral organoids	First human unguided organoids modeling brain regions and microcephaly	2013	Lancaster et al., 2013; Qian et al., 2016
Cortical organoids	Human three-dimensional neocortex with inside-out layering and progenitor dynamics	2013	Kadoshima et al., 2013
Hippocampal organoids	Self-organizing dorsomedial telencephalon producing hippocampal-like neurons	2015	Sakaguchi et al., 2015
Cerebellar organoids	Self-organization of polarized cerebellar tissue including Purkinje and granule cells	2015	Muguruma et al., 2015
Midbrain organoids	Midbrain-like organoids with functional dopaminergic neurons and neuromelanin	2016	Jo et al., 2016
Ventral forebrain organoids	Generated ventral spheroids and dorsal-ventral fusion models interneuron migration	2017	Bagley et al., 2017; Birey et al., 2017
Assembloids	Fusion of region-specific spheroids to model interregional integration	2017	Bagley et al., 2017; Birey et al., 2017
Immune-integrated organoids	Innate development of microglia-like cells within cerebral organoids	2018	Ormel et al., 2018
Vascularized organoids	*In vivo* transplantation leads to functional vascularization and maturation of grafts	2018	Mansour et al., 2018; Cakir et al., 2019
Brainstem organoids	Method to generate human brainstem organoids	2020	Eura et al., 2020

Early methodologies for generating neural organoids relied on the “default pathway” of inducing neural differentiation. That means when pluripotent stem cells do not receive external signals that induce mesendoderm (the embryonic tissue that generates both mesoderm and endoderm), intrinsic neural gene networks are preferentially activated. Pluripotent stem cells are cultured in the absence of patterning signals and form three-dimensional aggregates. After 2 weeks, neural tube-like structures can be observed within the cell aggregates, with defined cytoarchitecture and expression of neural genes, such as SOX2, ZO1, and PAX6, forming neural organoids. Then the organoids would be cultured in Matrigel-containing media or embedded in Matrigel to provide extracellular matrix support and mechanical signals that facilitate proper further tissue growth (Hemmati-Brivanlou and Melton, 1997; Muñoz-Sanjuán and Brivanlou, 2002; Eiraku et al., 2011; Lancaster et al., 2013).

To improve precise and reproducible neural organoid generation and to promote regional identities, culture signaling and environmental conditions in recent protocols have been standardized. There are two major directions. First, the culture conditions were controlled to promote forebrain identity by applying either signaling or anti-signaling molecules. For example, to inhibit non-neural lineage differentiation, TGFβ and BMP signaling were inhibited by dual SMAD inhibitors, such as SB431542 for TGFβ pathway inhibition and LDN-193189 or noggin for BMP pathway inhibition, and Wnt signaling was inhibited by DKK1 (Watanabe et al., 2005; Chambers et al., 2009). By doing so, more forebrain progenitor marker PAX6-positive ventricular zone-like structures appeared, with smooth apical surfaces and polarized cell arrangements in cortical organoids, mimicking brain development (Watanabe et al., 2005; Qian et al., 2016). In addition, signal molecules, such as Leukemia Inhibitory Factor (LIF), were applied at the certain period to promote the expansion of basal progenitors, especially outer radial glial cells. This promotes the formation of outer subventricular zone-like structures, which are the hallmark of human cortical development (Pollen et al., 2015; Andrews et al., 2023; Walsh et al., 2024).

Second, non-neural systems are integrated to facilitate *in vivo* physiological complexity. As the organoids grow, a solely neural composition faces challenges such as insufficient nutrients and a lack of immune infiltration. To overcome this, mesodermal lineages, especially those of the vascular and immune systems, are applied to brain organoids. To tackle metabolic constraints imposed by limited nutrient diffusion, researchers have incorporated endothelial cells to engineer primitive vascular networks, thereby significantly improving the metabolic sustainability of the organoid tissue (Mansour et al., 2018; Cakir et al., 2019). Regarding immune integration, the introduction of microglia has enabled simulation of critical neurodevelopmental processes, such as immune surveillance and synaptic pruning. This allows for deeper investigation into how the brain responds to developmental signals and external stresses (Abud et al., 2017; Ormel et al., 2018). To further enhance physiological realism, structural barriers, such as the blood-brain-barrier system, are established. By co-culturing endothelial cells with pericytes and astrocytes, this system recapitulates neurovascular coupling and selective molecular transport (Bergmann et al., 2018).

These improvements significantly reduced variability and established a robust platform for analyzing the molecular mechanisms of human brain growth.

## Milestones to simulate *in vivo* late brain development

A fundamental issue is whether these *in vitro* models can reliably reflect the complex and sophisticated progression of brain development. Early structural and microarray analysis demonstrated that that cortical organoids at approximately 3 months simulate the human fetal neocortex around 11 post-conceptional weeks (pcw) (Kadoshima et al., 2013). Single-cell transcriptomic mapping further showed that cerebral organoids at around 1–2 months closely mimic the gene expression programs of 12–13 pcw fetal neocortex, matching the genetic networks of radial glia, intermediate progenitors, and mature neurons (Camp et al., 2015), and 2.5-month-old cortical organoids strongly overlap with the mid-fetal prenatal brain at 19–24 pcw (Paşca et al., 2015). At the epigenetic level, in direct comparison to *in vivo* data, cortical organoids possess an intrinsic epigenetic clock that spontaneously undergoes global epigenetic remodeling, transforming tissue status from a fetal-like state to a postnatal-like state after long-term culture (over a year) (Gordon et al., 2021). This autonomous process suggests that mature molecular fates are pre-programmed within cells, validating long-term organoid culture as a high-fidelity platform for studying postnatal development and disease (Gordon et al., 2021).

In addition, the maturity of brain organoids can be evaluated by complex electrophysiological patterns. Cortical organoids in long-term culture can exhibit oscillatory waves mimicking EEG signals in human newborns, reflecting the formation of active functional networks (Trujillo et al., 2019). To further improve this maturity, recent studies have employed *in vivo* transplantation into rodent hosts, utilizing the host’s circulatory and sensory inputs to drive structural and functional refinement, thereby bridging the gap between *in vitro* potential and *in vivo* reality (Mansour et al., 2018; Revah et al., 2022).

## Generation of brain organoids with regional identities

The versatility of engineering techniques has enabled the successful modeling of distinct brain regions, each offering unique insights into human pathology. For example, organoids with dorsal forebrain identity can be generated by inhibiting TGFβ, BMP, and Wnt signaling. Within these dorsal forebrain organoids, the neural precursor cells inside undergo interkinetic nuclear migration, and the differentiated neurons migrate in an inside-out migration pattern, similar to the *in vivo* situation (Eiraku et al., 2008; Chambers et al., 2009; Kadoshima et al., 2013). By using Sonic Hedgehog agonists to induce ventral identity, ventral forebrain organoids, such as ganglionic eminence organoids, can be generated (Bagley et al., 2017; Birey et al., 2017).

Beyond forebrain organoids, protocols have been developed to generate organoids with other regional identities, such as the hypothalamus, midbrain, and cerebellum. Ventral diencephalic hypothalamic organoids can be generated by activating SHH pathways and inhibiting Wnt pathways and can secrete neuropeptides such as oxytocin and vasopressin, simulating the hypothalamus-pituitary axis and physiological responses to glucose, making them essential for studying metabolic disorders (Ozone et al., 2016; Burnett et al., 2017; Kasai et al., 2020; Huang et al., 2021). By activating the Wnt and SHH pathways, researchers have successfully generated midbrain organoids containing dopaminergic neurons and neuromelanin granules, which are pivotal for Parkinson’s disease studies (Kriks et al., 2011; Jo et al., 2016; Qian et al., 2016; Monzel et al., 2017; Smits et al., 2019). By mimicking isthmic organizer signaling with FGF19 and SDF1, researchers have generated cerebellar organoids capable of forming stratified layers of Purkinje and granule cells, providing a platform for modeling spinocerebellar ataxias (Muguruma et al., 2015; Ishida et al., 2016; Watson et al., 2018). These models are now robust enough to systematically assess neurological diseases via network activity analysis. Furthermore, to simulate natural brain development, such as long-range connectivity and neuronal migration, neural organoids can be physically assembled into assembloids. For example, the assembly of dorsal and ventral forebrain organoids enables investigation of interneuron migration and subtype differentiation (Uzquiano et al., 2022). Beyond brain-specific models, these assembloids provide an informative platform for circuit-level functional studies, including neuromuscular junctions and axonal projections (Birey et al., 2017; Andersen et al., 2020).

## General view of non-coding RNAs

**FIGURE 2:**
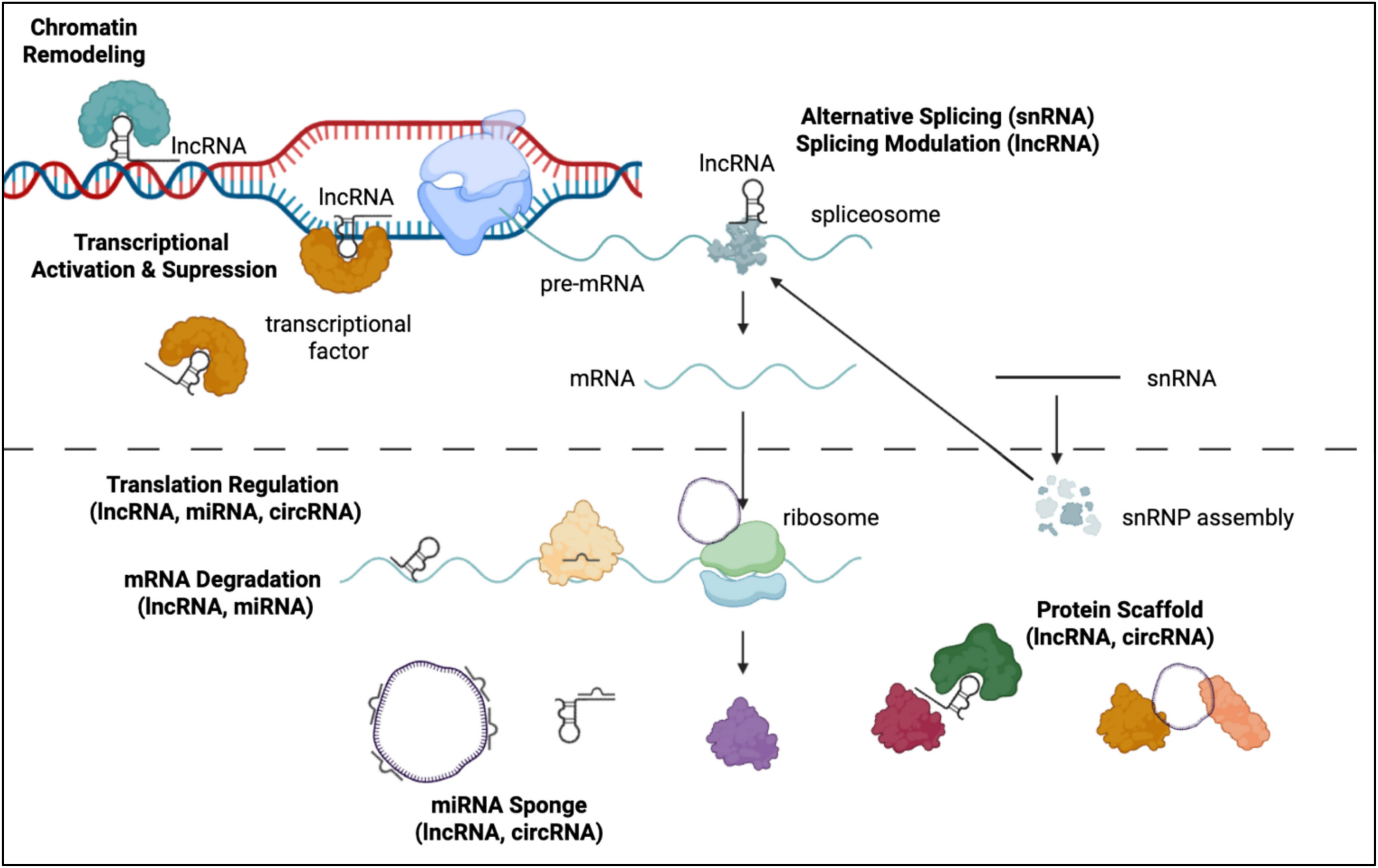
Summary of ncRNA functions in Central Dogma. LncRNAs participate in multiple regulatory levels, including chromatin remodeling at the DNA level, alternative splicing modulation, translation regulation, mRNA degradation, and acting as protein scaffolds. SnRNAs are essential components for alternative splicing, and miRNAs regulate mRNA stability and translation, but their activity can be repressed by lncRNAs and circRNAs acting as miRNA sponges. circRNAs can regulate translation and serve as both protein scaffolds and miRNA sponges. Created in BioRender. Chiang, P. (2026) https://BioRender.com/hbo7qyr.

Non-coding RNAs (ncRNAs) form a multi-layered regulatory landscape that operates beyond protein-coding genes, exerting control at the epigenetic, transcriptional, and post-transcriptional levels (Figure 2). Within this regulatory framework, ncRNAs are broadly categorized by size and functional mechanisms. Among the diverse ncRNA landscape, miRNAs and lncRNAs are recognized as master regulators of cell fate, while circRNAs and snRNAs provide critical layers of stability and molecular diversity essential for the complexity of human brain development.

MicroRNAs (miRNAs) are short, single-stranded RNAs that post-transcriptionally regulate gene expression by guiding Argonaute-containing complexes to complementary sequences in target mRNAs (Bartel, 2004). Complementary bindings between miRNAs and mRNAs typically occur within 3’ untranslated regions and result in translational repression or accelerated mRNA decay or both. Since a single miRNA regulates numerous mRNAs, and multiple miRNAs can target the same transcript, miRNAs are considered to act as network modulators with their complex targeting patterns. This allows buffering expression noise, coordinating gene programs, and setting thresholds for cell-fate transitions, thereby regulating multiple cellular functions, such as proliferation, differentiation, apoptosis, synaptic plasticity, and stress responses (Olde Loohuis et al., 2011; Hu and Li, 2017; Rajman and Schratt, 2017). In addition to their intracellular functions, recent work has shown that miRNAs also have cell-extrinsic functions. Researchers found that miRNAs can be selectively packaged into extracellular vesicles. Through exocytosis and endocytosis, miRNAs can modulate gene expression in recipient target cells (Valadi et al., 2007). Given their small size, extensive regulatory reach, and precise targeting, miRNAs have become valuable candidates for biomarkers and as therapeutic targets or agents in experimental systems (Bartel, 2004; O’Brien et al., 2018; Gebert and MacRae, 2019). While miRNAs are endogenous network modulators, small interfering RNAs (siRNAs) serve as their exogenous counterparts in experimental settings (Elbashir et al., 2001; Hannon, 2002). These short, double-stranded RNAs trigger sequence-specific gene silencing via the RNA interference (RNAi) pathway. Unlike the broad targeting of miRNAs, siRNAs are expected to achieve knockdown of single target transcripts, making them indispensable loss-of-function tools for dissecting functional gene requirements in development, stress response, and disease modeling, as well as developing therapeutic treatments. However, challenges regarding delivery methods, off-target effects, and the transient action of siRNAs remain to be overcome (Fire et al., 1998; Dana et al., 2017; Setten et al., 2019).

Long non-coding RNAs (lncRNAs) are a diverse class of transcripts that contain greater than 200 nucleotides. While the length of lncRNAs may be indistinguishable from conventional mRNAs and are often enriched in nucleus, many lncRNAs are exported to the cytoplasm to regulate mRNA stability and translation, or even serve as templates for the synthesis of functional micropeptides (Mattick et al., 2023). Currently, lncRNAs are found to regulate gene expression through multiple pathways. For example, lncRNAs may scaffold protein complexes to bring chromatin modifiers to target genomic loci, guide transcription factors and/or chromatin remodelers to modulate gene expression. They can also serve as molecular decoys or sponges that sequester proteins or microRNAs, therefore titrating the availability of these regulatory factors for their intended targets. In the nucleus, lncRNAs can influence chromatin architecture, enhancer–promoter looping, and splicing decisions, while, in the cytoplasm, they can control stability, translation, or localization of mRNAs (Song et al., 2021; Mattick et al., 2023). The high cell-type specificity of lncRNAs allows them to orchestrate developmental transitions and reinforce lineage commitment in response to extrinsic cues. By virtue of their modularity, these transcripts integrate diverse molecular layers, including epigenetic and RNA metabolic pathways, to ensure robust gene regulation in health and disease (Rinn and Chang, 2012; Quinn and Chang, 2016; Statello et al., 2021).

Circular RNAs (circRNAs) are covalently closed RNA species generated by back-splicing of exons or intronic sequences that resist exonuclease degradation, resulting in an extended half-life compared to their linear counterparts (Sekar and Liang, 2019; He et al., 2021). Multiple functions of circRNAs have been identified (Pamudurti et al., 2017). Similar to lncRNAs, circRNAs can act as sponges, binding and sequestering microRNAs or RNA-binding proteins, thereby modulating downstream molecular mechanisms. CircRNAs can also form protein scaffolds that bring enzymes and substrates together or influence the transcription and splicing of their linear host genes. Notably, circRNA expression is also in tissue- and stage-specific patterns, and certain circRNAs accumulate in the nervous system during maturation, such as Cdr1as, circHomer1, circSLC8A1, and so on (Hansen et al., 2013; Memczak et al., 2013; Rybak-Wolf et al., 2015; Gruner et al., 2016; Sekar and Liang, 2019; Jenks et al., 2025). Given their stability compared to single-stranded linear RNAs, circRNAs are expected to be mechanistic players in development and stress responses, potential biomarkers for disease, and therapeutic targets.

Small nuclear RNAs (snRNAs) form essential components of the spliceosome that process and regulate the splicing of pre-mRNAs. SnRNAs recognize splice sites and branch points through base-pairing interactions and, together with associated proteins, form the catalytic core, thereby enabling intron removal and exon ligation. By dictating splice-site choice and alternative splicing patterns, snRNAs profoundly influence isoform diversity, protein domain architecture, and temporal control of gene expression (Will and Lührmann, 2011; Matera and Wang, 2014).

Beyond these major classes, other specialized ncRNAs, such as small nucleolar RNAs (snoRNAs) and tRNA-derived fragments (tRFs), are increasingly recognized for their emerging roles in neural homeostasis and stress adaptation (Bratkovič et al., 2020; Yu et al., 2021; Wang et al., 2025).

## Recent studies on ncRNAs with neural organoids

A significant challenge in studying ncRNAs is the limitations of conventional two-dimensional (2D) cell cultures, which often lack histological and physiological complexity, thereby undermining their credibility. While 2D cultures provide foundational insights, they fail to recapitulate the intricate microenvironment and multicellular interactions critical for ncRNA function. In addition, cell movements and spatial organization in 2D differ from three-dimensional (3D) conditions *in vivo*. This gap prevents the thorough investigation of how ncRNAs regulate key brain developmental processes, such as neuronal migration and the formation of distinct cortical layers. To bridge this gap, human neural organoids have emerged as a sophisticated 3D model, enabling researchers to uncover the specific roles of ncRNAs in a context that closely mimics the developing human brain. The following sections highlight recent findings regarding the roles of ncRNAs, using neural organoids as a more representative modeling platform.

### miRNAs in neural organoids

MicroRNAs are essential regulators of neural cell identity and synaptic function, serving as highly sensitive indicators of developmental processes and environmental stress (Table 2). A recent study by Bahram Sangani et al. (2024) using MECP2-deficient human forebrain organoids identified notable upregulation of the chromosome 14 miRNA cluster C14MC and the miR-302/367 clusters, suggesting that these miRNA hubs contribute to neurodevelopmental aberrations. Given that MECP2 normally represses embryonic transcripts during maturation, its absence leads to developmental stalling, a state in which the failure to silence early-stage miRNA programs prevents cells from progressing toward a fully differentiated state (Bahram Sangani et al., 2024). Complementing these finding on cell fate, the dynamic turnover of miRNAs can also reflect the functional maturation, as demonstrated by Celiker et al. (2023) using human retinal organoids. They found that the miR-183/96/182 cluster, which acts as an immediate-early regulator of the visual cycle, is differentially regulated by distinct wavelengths of light (Celiker et al., 2023). This result recapitulates the findings *in vivo* adaptation mechanisms, thereby supporting the physiological maturity of retinal organoid systems.

**TABLE 2:** Summary of recent microRNA (miRNA) studies utilizing neural organoid models.

miRNA	Organoid model	Species	Major functions	References
let-7a-3p let-7a-5p let-7b-5p	Cortical organoids Retinal organoids	Human	Mediates PBDE-induced neurotoxicity	Lan et al., 2025
miR-124-3p miR-5683	Midbrain organoids	Human	Regulates mitotic control and organelle dynamics	Valente et al., 2025
miR-133b	Midbrain organoids	Human	Promotes dopaminergic neuron differentiation and maintenance	Valente et al., 2025
miR-873-5p	Midbrain organoids	Human	Linked to tauopathy as an early molecular hallmark	Valente et al., 2025
miR-219a-2-3p miR-219b-5p	Midbrain organoids	Human	Regulates mRNA transport and p53 signaling	Valente et al., 2025
miR-214	Midbrain organoids	Human	Involved in prodromal Parkinson’s pathophysiology	Valente et al., 2025
miR-145-3p	Midbrain organoids	Human	Parkinson’s disease biomarker	Valente et al., 2025
miR-138-2-3p	Midbrain organoids	Human	Associated with tau pathology	Valente et al., 2025
miR-204	Retinal organoids	Human	Maintains photoreceptor functionality	Arthur et al., 2025
let-7 family	Retinal organoids	Human	Promotes Müller glia and retinal neuron differentiation	Arthur et al., 2025
miR-21	Retinal organoids	Human	Modulates retinal endothelial function	Arthur et al., 2025
miR-21-5p miR-29a-3p miR-146a-5p	Cerebral organoids	Human	Inhibits NF-κB–mediated neuroinflammation in microglia	Li et al., 2025
mmu-miR-6991-3p	Brain organoids	Mouse	Reduces rotenone-induced neuronal death	Cui et al., 2025
miR-663a miR-663b miR-4273	Cerebral organoids	Human	Promotes progenitor differentiation into neurons	Trudler et al., 2025
miR-654-3p miR-451a	Retinal organoids	Human	Non-invasive biomarker of organoid differentiation and neurogenesis	Park et al., 2024
let-7b-5p	Retinal organoids	Human	Affects MAPK pathway genes to protect photoreceptors	Han et al., 2023
miR-122-5p	Retinal organoids	Human	Regulates proliferation and apoptosis via MAPK3	Han et al., 2023
miR-9	Retinal organoids	Human	Promotes neuronal differentiation	Celiker et al., 2023
miR-7	Retinal organoids	Human	Promotes photoreceptor differentiation	Celiker et al., 2023
miR-26a	Retinal organoids	Human	Controls neuronal differentiation	Celiker et al., 2023
miR-182-5p	Retinal organoids	Human	Light-induced and promotes photoreceptor proliferation	Celiker et al., 2023
miR-183-5p	Retinal organoids	Human	Light-induced and reduces photoreceptor oxidative stress	Celiker et al., 2023
miR-96-5p	Retinal organoids	Human	Light-induced and regulates photoreceptor synapse formation	Celiker et al., 2023
miR-204-5p miR-211-5p	Retinal organoids	Human	Light-induced and regulates photoreceptor synapse formation	Celiker et al., 2023
miR-145-5p	Retinal organoids	Human	Photostimulation-responsive and downregulated in inner nuclear layer	Celiker et al., 2023
miR-196a-5p	Retinal organoids	Human	Photostimulation-responsive and downregulated by light	Celiker et al., 2023
miR-205-5p	Retinal organoids	Human	Photostimulation-responsive and strongly downregulated by light	Celiker et al., 2023
miR-4516	Retinal organoids	Human	EV-enriched, targets photoreceptor and ganglion genes	Zhou et al., 2021
miR-4488	Retinal organoids	Human	EV-enriched, predicted to regulate TULP1	Zhou et al., 2021
miR-92b-3p	Retinal organoids	Human	EV-enriched, may regulate RGC axon guidance	Zhou et al., 2021
miR-204-3p	Retinal organoids	Human	EV-enriched, involved in RGC axon development	Zhou et al., 2021
miR-1298-5p	Retinal organoids	Human	Regulates neuronal projection and suppresses proliferation	Zhou et al., 2021
miR-99b-3p	Retinal organoids	Human	Regulates proliferation and retinoic-acid signaling	Zhou et al., 2021
miR-199a-3p	Cerebral organoids	Human	Upregulated in MeCP2-deficient progenitors and modulates AKT	Mellios et al., 2018

Beyond intracellular mechanisms, organoid studies show that retinal tissue coordinates development via extracellular vesicle (EV)-mediated cell-cell communication. For example, EVs secreted from human retinal organoids carry abundant miRNAs related to retinogenesis (Zhou et al., 2021). These vesicles would be internalized by retinal progenitor cells to regulate gene expression critical for photoreceptor differentiation, indicating specialized routes of cell-cell communication that support proper developmental progression (Zhou et al., 2021). In line with the importance of these secreted profiles, Park et al. (2024) define quality signatures of retinal organoids by profiling exosomal miRNAs. They identified has-miR-654-3p and has-miR-451a as being enriched in active stem cells with high proliferative and differentiation capacity, positioning these two miRNAs as non-invasive biomarkers for retinal organoid standardization and selection. Expanding on this diagnostic potential, a recent study further demonstrated the potential therapeutic strategy of injecting miRNA-containing exosomes derived from human iPSC-derived retinal organoids into the eyes of retinal degeneration rat models (Han et al., 2023). Their RNA-seq analysis revealed that these exosomal miRNAs primarily targeted and suppressed the MAPK signaling pathway, thereby exerting a neuroprotective effect that attenuated photoreceptor apoptosis and preserved visual function (Han et al., 2023).

The similar sensitivity that enables therapeutic signaling also renders these networks vulnerable to external interference. A recent study using human cortical and retinal organoids revealed the neurotoxicity of polybrominated diphenyl ethers (PBDEs) (Lan et al., 2025). Exposure to PBDEs disrupted specific miRNA–mRNA networks, particularly within the let-7 family, which plays a critical role in controlling neural differentiation timing. These results highlight the vulnerability of neurodevelopmental programs to environmental toxins, which can alter cell fate by selectively destabilizing miRNA-mRNA regulatory networks (Lan et al., 2025).

### lncRNAs in neural organoids

In neural organoids, lncRNAs have been shown to orchestrate neural lineage commitment and cortical programs by modulating chromatin states and gene expression (Table 3). For instance, the lncRNA SOX1-OT variant 1 (SOX1-OT V1) is involved in both dorsal cortical and ventral GABAergic neuronal differentiation. SOX1-OT V1 functions by binding and isolating the histone deacetylase HDAC10 away from the SOX1 promoter, thereby maintaining histone acetylation and enabling SOX1-driven neurogenesis (Xi et al., 2022). Similarly, PAUPAR, a lncRNA located adjacent to the transcription factor PAX6, physically interacts with PAX6 to guide its binding to neural gene targets and recruits the histone methyltransferase NSD1 to promote H3K36 methylation, collectively driving hESC-derived cortical differentiation (Xu et al., 2021).

**TABLE 3:** Summary of recent long non-coding RNAs (lncRNA) studies utilizing neural organoid models.

lncRNA	Organoid model	Species	Major functions	References
LINC01956	Glioblastoma organoids	Human	Promote glioblastoma growth and invasion	Deng et al., 2024
NEAT1	Forebrain organoids	Human	Downstream target of HHV-induced transposon activation	Feng et al., 2025
RP11-677M14.2	Brain organoids	Human	Cause synaptic damage in HIV context	Reis et al., 2024
IFNG-AS1	Brain organoids	Human	Sponges miR-21a-3p to promote neurogenesis	Fu et al., 2024
GIHCG	Glioblastoma organoids	Human	Promotes stemness and proliferation in glioblastoma stem cells	Hazra et al., 2023
LINC01563	Glioblastoma organoids	Human	Promotes stemness and proliferation in glioblastoma stem cells	Hazra et al., 2023
SOX1-OT V1	Cerebral Organoids	Human	Blocks HDAC10 to maintain SOX1 and neuronal differentiation	Xi et al., 2022
PAUPAR	Cerebral organoids	Human	Binds PAX6 to regulate cortical differentiation genes	Xu et al., 2021
lncGRS-1	Brain organoids	Human	Knockdown sensitizes glioma organoids to radiotherapy	Liu et al., 2020
TrEx lncRNAs	Dorsal forebrain organoids	Human, chimpanzee, orangutan, rhesus	Transient regulators of primate-specific cortical development	Field et al., 2019
HOXA10-AS	Glioblastoma organoids	Human	Promoting glioma proliferation, migration, and Hippo signaling	Isaev et al., 2021

Beyond these fundamental mechanisms, organoids offer a unique platform to explore the evolutionary expansion of the human cortex. An interesting finding from hESC-derived cortical organoids revealed that lncRNAs may exit the nucleus and enter the cytoplasm (An et al., 2023). They found that some loci within lncRNAs acquire cis-regulatory changes during evolution. When the ancestral lncRNAs accumulate those changes, they would receive enhanced splicing activity, and the U1 binding motifs, which are used to keep lncRNAs in the nucleus, may be reduced. As a result, lncRNAs may exit the nucleus and be subjected to the translation machinery (Lee et al., 2015; Yin et al., 2020; An et al., 2023). An et al. (2023) manipulated the human-specific *de novo* gene ENSG00000205704 in hESC-derived cortical organoids and identified its role in regulating neuronal maturation. They found that ENSG00000205704 knockout accelerated maturation, while overexpression delayed differentiation in hESC-derived cortical organoids. Also, ectopic expression in mice caused cortical enlargement (An et al., 2023). In addition to the molecular mechanism, recent cortical organoid studies have revealed numerous transiently expressed (TrEx) lncRNAs with highly specific spatiotemporal profiles during cortical differentiation. Their ectopic activation can alter neural gene programs, suggesting a role in primate-specific developmental progression (Field et al., 2019). In addition, some loci that once produced lncRNAs have evolutionarily transitioned into *de novo* protein-coding genes with measurable effects in organoids. These findings in brain organoids suggest lncRNAs and their derivatives as intrinsic regulators of the genomic landscape that defines human-specific brain architecture.

The regulatory reach of lncRNAs, as characterized in cerebral organoids, extends beyond intrinsic programs to non-cell-autonomous mechanisms, ranging from intercellular communication to pathogen-induced dysregulation. One emerging aspect is the role of exosomal lncRNAs. Fu et al. (2024) showed that exosomes derived from mesenchymal stem cells carrying IFNG-AS1 increased IFNG-AS1 levels in both brain organoid cultures and mouse prefrontal cortex. The upregulation of IFNG-AS1 levels acts as a molecular sponge for miR-21a-3p, thereby derepressing the PI3K/AKT pathway and promoting neurogenesis (Fu et al., 2024). Conversely, mature brain organoids are essential for modeling lncRNA dysregulation driven by neurotropic pathogens. For instance, Reis et al. (2024) demonstrated that HIV-1-induced neuroinflammation in brain organoids triggered the upregulation of the antisense lncRNA RP11-677M14.2. This transcript acts as a negative regulator of NRGN, a gene essential for synaptic function, suggesting a lncRNA-mediated mechanism for synaptic impairment during infection (Reis et al., 2024). Similarly, herpesvirus infection in human forebrain organoids triggered astrocyte-specific activation of the LINE-1 retroelement. This retrotransposon activity engaged long-range chromatin interactions to upregulate the lncRNA NEAT1 via altered enhancer–promoter looping (Feng et al., 2025). These organoid studies illustrate how lncRNAs function as critical mediators that translate viral insults into chronic inflammatory pathology in the 3D neural organoid system.

Glioblastoma organoids are also increasingly utilized to simulate tumor heterogeneity and the complex glioblastoma microenvironment. Regarding intrinsic growth, patient-derived organoids have identified lncRNAs, such as GIHCG and LINC01563 as critical drivers of glioma stem cell (GSC) proliferation and stemness (Hazra et al., 2023). Knocking down their expression would significantly impair tumor proliferation, migration, and stemness (Hazra et al., 2023). In addition, these models allow the investigation of the interplay with the immune microenvironment. For instance, the m^6*A*^-modified pseudogene transcript HSPA7 was shown to function by upregulating YAP1/LOX signaling in glioma stem cells to promote macrophage infiltration and modulate the glioblastoma microenvironment. Perturbing HSPA7 in patient-derived glioblastoma organoids was sufficient to alter immune interactions and improve therapeutic responses (Zhao et al., 2021).

In addition, a recent study utilizing CRISPRi screens in brain organoids under radiation stress identified the primate-conserved lncGRS-1 as a key regulator of radiosensitivity. Following validation by targeting lncGRS-1 in mature human brain organoids effectively reduced intratumoral growth and enhanced radiosensitivity (Liu et al., 2020). These studies collectively underscore the utility of the organoid platform for rigorous preclinical validation of lncRNA-based therapies.

### circRNAs in neural organoids

The transition from 2D cultures to 3D organoids enables the identification of circRNAs essential for tissue morphogenesis. A recent study profiled circRNA dynamics during hESC differentiation and identified circFAT3 as a pivotal, context-dependent regulator in 3D cortical organoids, while its knockdown in 2D cultures showed minor effects (Seeler et al., 2023). Specifically, circFAT3 deficiency led to the depletion of telencephalic radial glial cells and cortical neurons, thereby inducing severe structural defects. This divergence underscores that certain circRNAs are indispensable for proper forebrain architecture, a discovery that hinges upon the physiological complexity provided by the organoid system.

Beyond development, cerebral organoids subjected to oxygen–glucose deprivation (OGD) have been used to simulate the physiological stress of ischemic stroke. Researchers discovered that circFGFR2 is significantly upregulated in astrocytes, where it drives inflammatory damage by promoting NLRP3-dependent pyroptosis, suggesting that targeting specific circRNAs could offer novel therapeutic avenues for stroke (Pang et al., 2025). Similarly, in the context of genetic disorders, retinal organoids have been employed to model a rare X-linked dystrophy. This study linked structural variants at the LINC00632 locus to downregulation of CDR1as, which, in turn, dysregulated neuronal miR-7 targets (Gardner et al., 2025). Such findings illustrate how organoids can faithfully simulate the complex RNA regulatory perturbations characteristic of human genetic diseases.

In addition, the utility of organoids extends to neuro-oncology, bridging the gap between basic biology and clinical applications. In a glioblastoma organoid model, circPTTG1IP is identified as a critical biomarker for drug resistance. Mechanistic analysis showed that circPTTG1IP modulates the NAMPT/NAD+ metabolic axis, thereby directly influencing cancer cell survival and sensitivity to combined therapies like Olaparib and FK866 (Sha et al., 2025). Collectively, these studies underscore that circRNAs are active, functional factors in the human brain development and disease, presenting a new frontier in understanding neural health and disease using the organoid platform.

### snRNA in neural organoids

Human cortical organoids provide a unique window for investigating how spliceosome dysregulation leads to severe brain malformation, such as Taybi-Linder Syndrome (TALS). TALS is defined by mutated U4atac snRNA. Nonetheless, recent organoid research has uncovered a pathological mechanism to between this U4atac snRNA and the RTTN protein gene (Guguin et al., 2024). They found RTTN variant cells fail to form proper ventricular zone-like structures. Further mechanistic analysis revealed that this defect originated from impaired centrosomes and cilia, which, crucially, is the same cellular machinery targeted by U4atac snRNA-dependent splicing. This study bridges the gap between disparate genetic triggers, demonstrating that whether the primary insult resides in a non-coding snRNA or a structural protein, they can disrupt the same developmental program. Such insights underscore the unique capacity of human cortical organoids to simulate the intricate regulatory overlaps essential for proper brain cytoarchitecture (Guguin et al., 2024).

## Remaining issues confronted while investigating ncRNAs in neural organoids

While the functions of ncRNAs have begun to unravel in recent decades, the whole picture of non-coding RNAs, including lncRNAs, miRNAs, and siRNAs, in regulating brain development remains hindered by the limitations of traditional research models. One of the primary obstacles is the species-specific differences. Human brain development lasts an exceptionally long period of progenitor expansion, including expanded progenitor subtypes (e.g., outer radial glia), neurogenesis, and maturation, compared to non-human primates and other mammals (Prodromidou and Matsas, 2021). These differences are not limited to the developmental timeline, but also involve cellular diversity and the molecular environment in which ncRNAs function (Bocchi et al., 2021). Importantly, the number and types of ncRNAs increase significantly in higher organisms, suggesting that a large portion of this expanded ncRNA repertoire may contribute to human-specific neurodevelopmental processes (Morales-Vicente et al., 2024). Whether the evolutionarily conserved ncRNAs play conserved roles or have evolving roles during this long process compared to non-human species remains unknown (Qureshi and Mehler, 2012). In addition, increasing evidence has shown that distinct neurodevelopmental features essential to cortical expansion are largely driven by human-specific regulatory networks that standard laboratory rodents do not intrinsically possess (Zimmer-Bensch, 2019; Prodromidou and Matsas, 2021; Lin et al., 2026). These raise important questions: which platforms are suitable, how far non-human findings can be translated to humans, and whether the expanded ncRNA repertoire in higher organisms plays roles that simply cannot be modeled in other species.

Furthermore, while the number of protein-coding genes has not expanded proportionally with brain complexity across mammals, the ncRNA transcriptome has expanded significantly in higher organisms (Mattick, 2011). This expansion coincides with the increasingly complex brain system in higher-order animals, such as primates, suggesting the evolving ncRNA system may provide a fundamental basis of intricate regulatory networks during brain evolution. Thus, the magnitude of ncRNA-mediated regulation in humans would provide precise spatiotemporal control, layering epigenetic and transcriptional instructions necessary for human brain development. For instance, primate-specific transiently expressed lncRNAs regulate cell-type-specific gene networks essential for human cortical differentiation (Field et al., 2019), and human-specific *de novo* genes with lncRNA origins actively dictate the timeline of neuronal maturation (An et al., 2023). Such examples highlight that the magnitude of ncRNA-mediated regulation in humans goes beyond what current animal models can recapitulate, posing significant challenges in deciphering the functional impact of ncRNAs uniquely expressed or differentially regulated in the developing human brain.

Despite the promise of neural organoids, these systems still harbor limitations that can profoundly affect ncRNA transcriptomic readouts and warrant careful consideration. For instance, the absence of a fully functional vascular network in organoid cultures would restrict nutrient and oxygen diffusion, creating a non-physiological hypoxic microenvironment. This would trigger metabolic stress responses, including endoplasmic reticulum stress and a shift toward glycolysis-dependent metabolism (Nallamshetty et al., 2013; Bhaduri et al., 2020). Mechanistically, hypoxia-inducible factors (HIFs) are activated under these conditions, directly influencing oxygen- or stress-sensitive miRNAs or lncRNAs, such as miR-210 and other stress-responsive ncRNAs, leading to abnormal upregulation and potentially biasing the observed ncRNA profiles away from physiological states (Nallamshetty et al., 2013). In addition, the absence of physiological inputs such as sensory stimuli and immune infiltration may prevent activity-dependent ncRNA transcriptomes from reaching full maturation (Ormel et al., 2018; Revah et al., 2022). Therefore, while neural organoids are indispensable, these limitations need to be carefully considered when investigating ncRNA functions to avoid misinterpretation.

Additionally, researchers face technical hurdles in tracking temporal dynamics. Monitoring changes over time in human tissue is difficult, complicating the identification of critical developmental windows. Besides, delivering therapeutic siRNAs into complex tissues remains a major challenge. Traditional toxicology screenings also tend to overlook subtle molecular changes, often detecting only obvious cell death rather than the subtle rewriting of RNA programs caused by environmental toxins. Given the aforementioned issues, the use of organoids to investigate ncRNAs has become increasingly essential.

## Conclusion and future perspectives

The integration of non-coding RNA (ncRNA) research with neural organoid technology has established a powerful platform to decipher the functional complexities of the human non-protein-coding genome. By providing a physiologically relevant 3D context, organoid models have validated that ncRNA functions are inherently context-dependent, ranging from lncRNA-mediated epigenetic remodeling and exosomal communication to the dynamic switching of miRNAs during differentiation. Furthermore, these systems have illuminated the indispensable roles of circRNAs and snRNAs in maintaining spliceosomal integrity and orchestrating the precise layering of the human cortex, which are the features difficult to recapitulate in traditional 2D or animal models. The capacity of neural organoids to represent these intricate regulatory networks offers more than mechanistic insights. It is expected to offer a rigorous framework for preclinical target validation. As we move toward a more “cell-free” therapeutic era, harnessing organoid-derived exosomal ncRNAs and CRISPR-based modulation of lncRNA hubs may pave the way for correcting the molecular defects underlying neurodevelopmental and neurodegenerative conditions. In conclusion, neural organoids remain an unparalleled platform for decoding the non-coding landscape of the human brain, providing the structural and physiological context necessary to unlock the full potential of ncRNAs in neurodevelopmental medicine.
